# Response of pumas (*Puma concolor*) to migration of their primary prey in Patagonia

**DOI:** 10.1371/journal.pone.0188877

**Published:** 2017-12-06

**Authors:** Maria L. Gelin, Lyn C. Branch, Daniel H. Thornton, Andrés J. Novaro, Matthew J. Gould, Anthony Caragiulo

**Affiliations:** 1 Department of Wildlife Ecology and Conservation, and School of Natural Resources and Environment, University of Florida, Gainesville, Florida, United States of America; 2 School of the Environment, Washington State University, Pullman, Washington, United States of America; 3 Programa Estepa Patagónica y Andina, INIBIOMA-Universidad Nacional del Comahue-CONICET, Wildlife Conservation Society, Junín de los Andes, Neuquén, Argentina; 4 Department of Biology, New Mexico State University, Las Cruces, New Mexico, United States of America; 5 Sackler Institute for Comparative Genomics, American Museum of Natural History, New York, New York, United States of America; Panthera, UNITED STATES

## Abstract

Large-scale ungulate migrations result in changes in prey availability for top predators and, as a consequence, can alter predator behavior. Migration may include entire populations of prey species, but often prey populations exhibit partial migration with some individuals remaining resident and others migrating. Interactions of migratory prey and predators have been documented in North America and some other parts of the world, but are poorly studied in South America. We examined the response of pumas *(Puma concolor)* to seasonal migration of guanacos *(Lama guanicoe)* in La Payunia Reserve in northern Patagonia Argentina, which is the site of the longest known ungulate migration in South America. More than 15,000 guanacos migrate seasonally in this landscape, and some guanacos also are resident year-round. We hypothesized that pumas would respond to the guanaco migration by consuming more alternative prey rather than migrating with guanacos because of the territoriality of pumas and availability of alternative prey throughout the year at this site. To determine whether pumas moved seasonally with the guanacos, we conducted camera trapping in the summer and winter range of guanacos across both seasons and estimated density of pumas with spatial mark–resight (SMR) models. Also, we analyzed puma scats to assess changes in prey consumption in response to guanaco migration. Density estimates of pumas did not change significantly in the winter and summer range of guanacos when guanacos migrated to and from these areas, indicating that pumas do not follow the migration of guanacos. Pumas also did not consume more alternative native prey or livestock when guanaco availability was lower, but rather fed primarily on guanacos and some alternative prey during all seasons. Alternative prey were most common in the diet during summer when guanacos also were abundant on the summer range. The response of pumas to the migration of guanacos differs from sites in the western North America where entire prey populations migrate and pumas migrate with their prey or switch to more abundant prey when their primary prey migrates.

## Introduction

Large-scale ungulate migrations associated with changes in availability of resources and risk of predation are widespread phenomena that influence mammal community composition, prey abundance for top predators, and fitness of predators [1–3]. In many systems with ungulate migrations, not all individuals migrate, but even partial migrations can result in large spatial changes in prey biomass [4–7]. Predators track prey movements by undertaking migrations with them, or they can modify their foraging behavior (e.g., exhibit prey switching or incorporate additional prey in the diet when large numbers of their prey migrate [3,8–10]). Some predators also hold territories throughout the year near den sites and then make long distance foraging trips to areas with migratory herbivores once these herbivores have moved away from the territory [11,12]. Predator responses to prey migration have diverse effects at the community level. For example, if predators move with prey, the distribution of carcasses for scavengers and decomposers also shifts [13,14]. On the other hand, if predators remain relatively stationary and switch prey or broaden their diet in response to migration, they may have significant impacts on populations of alternative prey [15,16]. Migration of native prey also may affect frequency of predation on livestock and increase human-predator conflict if livestock serve as alternative prey sources for predators [10,12,17].

Behavioral responses of carnivores to prey migration have been documented for a variety of species and ecosystems in North America (e.g., [9,12,18]), but both herbivore migrations and predator responses to these migrations are poorly studied in South America. One predator that spans both of these continents is the puma (*Puma concolor*), which has the largest geographic range of any mammal in the western hemisphere and is a top predator from Canada to southern South America. Although pumas rely on non-migratory prey in much of their range, studies in North America have reported migration by pumas [18] and prey switching [9] in systems where their primary prey, large ungulates, migrate.

In Patagonia in southern South America, pumas prey heavily on the largest native ungulate, the guanaco (*Lama guanicoe*), in areas where this species is still common [19–21]. Although guanaco populations have declined throughout their range and movement patterns likely have been altered, the longest known terrestrial migration for mammals in South America is seasonal migration of guanacos in northern Patagonia, Argentina [22,23]. The ecological impacts of this migration are unknown. In this study, we examined the response of pumas to this guanaco migration, which occurs in La Payunia Reserve, the protected area with the largest population of guanacos (> 25,000 individuals) across the entire range of the species. Approximately two-thirds of the population is migratory; other individuals are resident in the summer or winter range throughout the year [23–25]. Although La Payunia Reserve is one of the few sites in South America where long-distance ungulate migrations are well documented, shorter migrations and other seasonal movements of guanacos are known to occur in other areas and likely were more common in the past when guanaco populations were large and barriers to movement were less common [22,26,27].

We hypothesized that pumas respond to migration of large numbers of guanacos by switching to alternative prey, or by consuming more alternative prey along with resident guanacos, rather than migrating with guanacos because pumas generally are territorial, have broad food habits [28,29], and alternative prey are common in La Payunia and available throughout the year. We expected pumas to include more livestock as well as native prey in their diet when guanacos migrated. Ranching of goats and cattle occurred within the reserve and on neighboring lands, and puma predation on livestock was a significant source of conflict. Local herders reported a higher frequency of puma attacks on livestock in areas where guanaco abundance was low, but this predation has not been explicitly linked to the guanaco migration [24].

To test our hypothesis, we examined puma density in summer and winter ranges of guanacos and analyzed diet of pumas from scats to determine whether pumas shift their diet when guanacos migrate. If, in contrast to our hypothesis, pumas move with guanacos, we expected to see changes in puma density that paralleled guanaco migration. We estimated puma density from camera-trapping data with spatial mark-resight models. Spatial mark-resight models are a new tool developed to overcome important limitations of capture-recapture models related to the need to better define effective sampling area and, at the same time, incorporate unidentified individuals in density estimates [30–33].

## Materials and methods

### Study area

**Fig 1:**
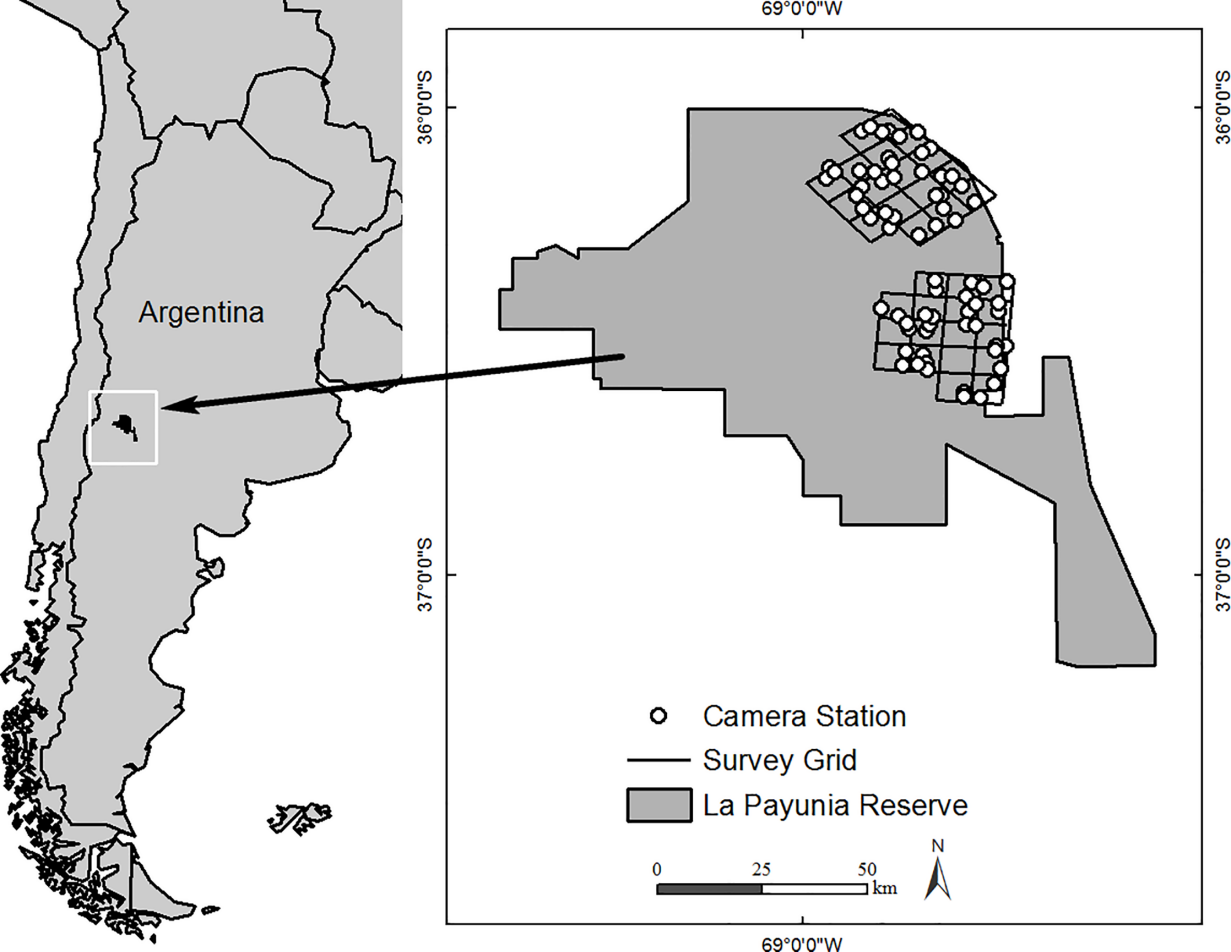
Location of La Payunia Reserve in Mendoza Province, Argentina, boundaries of the reserve, and layout of north and south grids within the reserve. Camera stations are marked with dots within the grids. The north grid is located in the summer range of migratory guanacos, and the south grid is located in their winter range.

Our study was conducted in La Payunia Reserve (36°10’S, 68°50’W, Fig 1), a provincial reserve (6641 km^2^) in northern Patagonia, Mendoza Province, Argentina. The northern part of the reserve is characterized by a high density of ancient volcanoes (1400–2000 m above sea level) dispersed across broad plains, and the south contains open plains and lower elevations (1140–1400 m above sea level). Guanacos occur throughout the reserve and migratory herds move either to the west or the south for winter [34]. Our study focused on the eastern part of the reserve where migratory guanacos winter in the south and move up to 70 km to the north in summer [S1 Fig]. Alternative native prey for pumas in La Payunia Reserve include the plains vizcacha (*Lagostomus maximus*), southern mountain vizcacha *(Lagidium viscacia)*, lesser rhea *(Rhea pennata)*, Patagonian mara (*Dolichotis patagonum)*, European hare *(Lepus europaeus)*, pichi armadillo *(Zaedyus pichiy)*, elegant crested tinamou *(Eudromia elegans)*, and a variety of small mammals [e.g., tuco-tucos, (*Ctenomys spp*.*)*, southern mountain cavy (*Microcavia australis*), and Cricetid rodents] [20,35,36]. Smaller carnivores also occasionally may be consumed by pumas [e.g., *S*outh American grey fox *(Lycalopex griseus*), Geoffroy’s cat *(Leopardus geoffroyi)*, and pampas cat *(Leopardus colocolo)*]. Vegetation throughout the reserve (~ 50% cover) comprises small shrubs interspersed with grasses. Dominant species include: *Neosparton aphyllum*, *Chuquiraga erinacea*, *Larrea divaricata*, *Cassia aphila*, *Panicum urvilleanum*, *Poa* spp., and *Stipa* spp. [25,37]. Annual precipitation averages 255 mm, occurring mostly during the summer months, and mean seasonal temperatures range from 6°C in winter to 20°C in summer [38].

Approximately 55% of La Payunia Reserve is under government protection. The rest is managed by private ranchers and herders whose main economic activity is extensive livestock ranching, principally cattle and goats [24,37]. Despite the protected status of the area, pumas and other predators occasionally are killed in response to livestock depredation.

### Camera-trap surveys

We conducted field surveys with remotely-triggered cameras (Browning BTC-5 HD Strike Force) for 11,442 camera-station trap days. Sampling occurred on two grids from 23 July 2015–3 January 2016, which represents mid-winter 2015 to mid-summer 2016. For our study, we considered winter as 23 July– 5 October 2015 and summer as 6 October 2015–3 January 2016. Each grid was approximately 720 km^2^. This grid size is among the largest survey areas used to estimate puma density with camera traps [39–43]. One grid was located in the summer range of migratory guanacos (north grid, Fig 1) and one grid was located in the winter range (south grid). Each grid was divided into 17 cells of 6 x 6 km^2^. We placed two camera stations (hereafter stations) in each cell, except in one cell in the south grid where we could not get permission from the landowner to access his land and placed 4 stations in the adjacent cell to the west. Also, we placed one extra camera station in the north grid (total stations: north grid, 35; south grid, 34). Stations within a cell were separated by at least 1 km and were placed in sites pumas were likely to use (e.g., drainages and rocky outcrops), where possible. Two cameras were oriented facing each other at each station to obtain photographs of both sides of animals to facilitate identification of individuals from natural markings and for redundancy in case of camera failure [32,44,45]. To increase the quantity and quality of photographs of pumas in each station, we placed perfume (Obsession by Calvin Klein) as an attractant on a stake approximately 50 cm tall between the two cameras [46,47]. Cameras were programmed to function 24 h/day with a minimum interval of five seconds between photographs. Data from photographs (camera number, date and time) were extracted using the camtrapR package [48] implemented in R.

Although pumas do not have as obvious marks as some felids that are surveyed with cameras [e.g., jaguars (*Panthera onca*) and tigers (*P*. *tigris*)], Kelly et al. [39] and subsequent studies [e.g., 41,43,49] demonstrated that individual pumas can be reliably identified based on natural marks such as ear nicks, scars, and kinked tails. However, given that SECR analyses can incorporate data for images of known and unknown individuals and some pumas were photographed by only one camera from long distances in this open landscape, we took a conservative approach and assigned individual identification only to pumas that were photographed clearly from both sides (43% of the independent records). Photographs were identified by the lead author. The short interval programmed between photographs often resulted in multiple images of the same individual from both sides and facilitated identification of these individuals.

Our field research protocols met Guidelines of the American Society of Mammalogists [50]; we did not manipulate any pumas, nor did we modify the habitat (other than collection of scats as approved by the Dirección de Recursos Naturales Renovables, Mendoza, Resolución n° 265). Methods for this project were approved by University of Florida’s Institutional Animal Care and Use Committee (IACUC Protocol Number 201508868).

### Puma density estimates

To estimate puma density, we used spatial mark-resight models (SMR) within a likelihood analysis framework implemented in the R package ‘secr’ [33]. Spatial mark-resight models are a new extension of spatially-explicit capture-recapture models (SECR; [30,31]) that allow density to be estimated in cases where only part of the population can be identified to the individual level [32]. Unidentified individuals are incorporated in the SMR modeling and treated as independent of identified sightings [33]. Another advantage of both SMR and SECR over earlier non-spatial capture-recapture models is that spatial data on captures informs density estimates. The spatial coordinates of the camera traps where individuals are captured provide information on the location of the animal’s activity center [31,32]. The probability of an individual being captured is modeled as a function of distance between the survey location (i.e., camera) and the animal’s activity center. This approach addresses a primary criticism of other methods of estimating density from camera trapping which is the need to calculate a reliable effective trapping area [42,51].

Input files for SMR include information on animal captures from photographs, trap deployment (i.e., camera locations and days the cameras were active), and a habitat mask. For photographs of identified individuals, we created encounter histories that included individual animal identification, encounter occasion, and camera station in which the individual was detected. For unidentified individuals, encounter histories were similar but all captures were registered with the same identification name (UN). We used one record per day per station as an independent record for identified individuals and also for unidentified pumas. We collapsed daily camera-trap data into 10 time intervals (i.e., 10 encounter occasions each comprising a 19-day sampling period), prior to fitting Poisson count models in SECR [33,52]. The time interval used for encounter occasions has no effect on parameter estimates (e.g., density) with Poisson count models (Efford, personal communication), but using fewer occasions significantly reduces processing time. The habitat mask, which consists of equally spaced point locations for the study area, defines spatial limits for modeling and can be used to build density models with habitat covariates [33]. The mask is constructed by placing a buffer around the outer-most camera locations with buffer width defined by a distance that ensures that animals with home range centers outside that distance have a negligible chance of being detected on the camera grid [33,51]. We used a buffer of 12 km based on the R function ‘suggest.buffer,’ which determines a suitable buffer width from movement of animals between cameras. We checked that this buffer was sufficiently large (i.e., encounter rate of pumas was near 0 at the edge of the buffer) using function esa.plot ([33], S2 Fig). We generated potential home range centers within this buffered area as a regular grid of points with 2-km spacing.

Spatial mark-resight models provide 3 model parameter estimates: density (D), expected number of detections if the home range center were located at the trap (i.e., encounter rate, λ0), and a spatial scale parameter (σ) that relates decline in encounter rate with distance from the animal’s activity center [32,53]. We fit models for λ0 with a constant encounter rate (.) and several covariates including a time factor model (‘t’, where encounter rate varies by sampling occasion), a site learned response model (‘k’, where encounter rate at the site changes after an animal is recorded at that location), and a learned response model (‘b’, where the encounter rate of an individual at all sites changes, dependent on previous capture; [33]; S1 Table). A learned response may be most likely to occur in studies that capture animals in traps, but could occur in our study if the perfume added as an attractant influenced puma behavior. We did not include sex in our analysis because sex of individuals often could not be determined. We added a covariate (site) to determine if location of stations (drainages and rocky outcrops versus open plain) influenced encounter rate. We expected cameras in drainages and rocky outcrops to have higher detection probability because these sites have features that would channel pumas between the two cameras.

To examine whether density of pumas changed seasonally in response to guanaco migration, we fit a multi-session model where each session was a different combination of trapping grid and season (north grid_summer, north grid_winter, south grid_summer, south grid_winter) and compared this to a base model with no factors using Akaike’s Information Criteria corrected for finite sample size (AICc; [32,33,51]). The multi-session model also was fit with the covariates for λ0. Each session represented 5 encounter occasions (i.e., 95 trapping days) and likely fulfilled the assumption of a demographically closed population during the survey. As in other studies, we assumed that effects of mortality, immigration, and emigration were negligible because sessions were short relative to the lifespan and territory tenure lengths of pumas [29,39,40,49,54]. The base model generated density estimates with 190 trapping days (i.e., all 10 occasions) and may violate assumptions of demographic closure.

### Collection and identification of puma scats

Puma scats were collected along standardized transects as well as opportunistically in drainages and other suitable puma habitat throughout the study area. Collection began prior to camera trapping but occurred in areas where grids eventually were established. Eight 4-km transects were walked in the area occupied by each camera grid repeatedly in mid-late summer 2015 (January-April) and winter 2015 (May-September). From mid-winter 2015 through mid-summer 2016 (July 2015—January 2016), we also searched for puma scats along the paths used to install and check cameras. Puma scats were distinguished from those of other sympatric predators (e.g., foxes and small cats) in the field by their size and shape, and identification was later confirmed for all samples by DNA analysis at the American Museum of Natural History using the methods outlined in Caragiulo et al. [55]. A small piece of each sample was used for DNA analysis, and the remainder of the samples was washed and sundried for lab analysis of prey. Prey items in scats were identified to species using keys for hair of Patagonian mammals [56] and by comparing hairs to known hair samples obtained from specimens in the Florida Museum of Natural History and from dead animals found in the reserve. Identification of hairs was based on scale and medulla patterns [57].

### Diet analysis

From a total of 129 puma scats collected for prey analysis, 43 scats were fairly fresh (e.g., retained black or brown color and often odor, and exhibited no apparent degradation) and thus were identified as being deposited during the period when guanacos were abundant or scarce in the area. We only analyzed scats collected from the north grid (*n* = 39) because the sample size from south grid was very small (*n* = 4). Scats were found throughout the north grid and, therefore, were assumed to broadly represent the diet of the pumas in this area (Table 1). We calculated frequency of occurrence per scat for all food items (i.e., percentage of scats containing a particular food item), which provides information about rare as well as common items in the diet [58]. To test for changes in the diet of pumas with the guanaco migration, prey items in scats were divided in two major groups: guanacos and alternative prey, which included small and medium-sized mammals and livestock. Then we compared the frequency of occurrence of items consumed in the two groups between the period when guanacos were abundant on the north grid (summer) versus more scarce (winter) using a Fisher’s Exact test [59].

**Table 1:** Results of camera trapping and density estimation for pumas on two grids in La Payunia Reserve, Argentina.

	n*	ID	non-ID	Survey duration (days)	Survey effort (trap days)	Density estimate (95% CI)
North Grid						
Summer	11	39	51	95	3002	4.3±1.1 (2.6–7.1)
Winter	9	20	39	93	3087	3.6±0.9 (2.1–6.0)
South Grid						
Summer	5	12	20	94	2944	2.2±0.8 (1.1–4.5)
Winter	4	11	16	94	2409	1.8±0.7 (0.8–4.0)

n* = total number of pumas identified as individuals, ID = number of independent records in which pumas were identified as individuals, non-ID = number of independent records that were not identified to the level of individual, density estimate (pumas/100 km^2^ ± *SE*) and 95% confidence intervals (CI). Kittens were excluded from analyses.

## Results

### Photographic records of pumas

We obtained 149 independent records of pumas on the north grid from which we identified 12 individuals (Table 1). These pumas occurred in 59 of 149 records. For the south grid, we obtained 59 records of pumas and identified 6 individuals. These pumas occurred in 23 of 59 records. The mean number of recaptures per individual was: north grid, 3.8 (range, 1–9); south grid, 3.0 (range, 1–5). Only one identified puma, a male as evidenced by the scrotum, was detected in both the north and south grids. The mean of maximum distances moved between cameras by individual pumas was: north grid, 6.2 km (range, 1.6–21.0); south grid, 6.8 km (range, 2.2–11.1).

### Puma density

We detected no change in puma density over space and time as would be expected if pumas moved with north-south migration of guanacos. However, heterogeneity in density estimates was supported by the lower AICc of the multi-session SMR model [D(session) λ0(site), σ (.); AICc = 1432, λ0 = 0.02, σ = 754, number of parameters (K) = 7] compared to the base model [D(.) λ0(site), σ (.); AICc = 1532, λ0 = 0.01, σ = 771, K = 4]. Density estimates were highest in the northern part of our study area in summer when most guanacos were in the area, as predicted if pumas were tracking guanacos, and densities were about 16% lower in winter when many guanacos had migrated south to the winter range (Table 1). However, in the southern part of the study area, estimates of puma density followed the same seasonal trend with a 18% decline in winter, opposite of the expected pattern based on the guanaco migration. The northern part of the study area had consistently higher density estimates than the south, and these differences were more pronounced than seasonal changes in density within either area (Table 1).

The expected number of detections for an individual at a particular site was more than 4 times higher for camera stations in drainages and rocky outcrops than in open habitat (0.083 versus 0.019), and both the multisession model and the base model were improved by including site as a covariate for capture probability (S1 Table). Models with other covariates for capture probability were not competitive. The 12-km buffer incorporated around camera stations was sufficient; effective sampling area reached an asymptote by 7 km and expected number of detections declined to near 0 well before the edge of the buffer (S2 Fig).

### Puma diet

In contrast to our prediction, pumas did not switch to alternative prey or broaden their diet during winter when migrating guanacos moved away from the north grid. Puma diet was broadest in summer when guanacos also were most abundant, and thus exhibited a trend in the opposite direction from our predictions (Fisher’s Exact test, *n* = 49, *P* = 0.072, *d*.*f*. = 1). Remains of guanacos were found in 85% of puma scats during the winter and only in 64% of scats during the summer. Three alternative prey species were recorded in winter and 8 were recorded in summer, from a total of 16 and 33 items, respectively (Table 2). Overall, guanacos were the most common item in scats (71.8% of the scats), followed in order of importance by plain vizcachas and European hares, dwarf armadillos, and small rodents. Livestock remains (cow, *Bos Taurus*, and goat, *Capra hircus*) were found only in one scat in summer and one in winter.

**Table 2:** Frequency of occurrence and counts of prey items in puma scats.

	Frequency of occurrence	
Prey	Winter	Summer
Main Prey		
Guanaco *(Lama guanicoe)*	85.7 (12)	64.0 (16)
Alternative Prey		
Plains vizcacha *(Lagostomus maximus)*	0	20.0 (5)
European hare *(Lepus europaeus)*	14.3 (2)	12.0 (3)
Dwarf armadillo *(Zaedyus pichiy)*	7.1 (1)	4.0 (1)
Geoffroy's cat *(Leopardus geoffroyi)*	0	4.0 (1)
Southern mountain cavy *(Microcavia australis)*	0	4.0 (1)
Other small mammals	0	8.0 (2)
Cow (*Bos taurus*)	7.1 (1)	0
Goat *(Capra hircus)*	0	4.0 (1)
Unidentified mammal	0	12.0 (3)

Prey items were identified in puma scats in summer (*n* = 25 scats) and winter (*n* = 14 scats) in the north of La Payunia Reserve, Mendoza, Argentina, which serves as summer range for migratory guanacos. Numbers in parentheses correspond to counts of prey items.

## Discussion

Our results indicate that pumas are not following the migration of guanacos in La Payunia Reserve or switching to alternative prey when guanacos are less abundant. Density estimates of pumas did not increase significantly in the winter and summer ranges of guanacos when guanacos migrated to these areas. Pumas included a greater variety of species in the diet in the summer when guanacos were abundant on the summer range. Given that the corners of our two grids were only about 7 km apart, we expected home ranges of some pumas to span parts of both grids (i.e., the summer and winter ranges of guanacos). One of the 18 pumas individually identified was detected in the north grid in late September and then was recorded 45 km away in the south grid in December, but the movement of this animal was in the opposite direction expected if this puma were following the guanaco migration.

Although the seasonal migration of guanacos in La Payunia is massive, as in many other migratory ungulate populations, part of the population does not migrate [4,7,24]. Resident guanacos, together with some alternative prey, apparently provided sufficient food so that pumas did not have to track migration of their primary prey. Also, pumas may specialize in killing guanacos in La Payunia, as has been reported for southern Chile [60,61], and be efficient at taking guanacos even when guanaco density declines. Most alternative prey consumed by pumas in this study were small relative to guanacos, and thus would comprise a small portion of the overall biomass in the puma diet. Consumption of these species could reflect opportunistic foraging behavior of pumas, prey selection, or behavior and dynamics of prey [36,61–64]. Individual pumas or particular sex and age classes sometimes specialize on non-ungulate prey [61,65]. During this study, the plains vizcacha, cavy, and several other species were found in puma scats only in summer. Plains vizcachas were the most common alternative prey in summer. Seasonal predation on this species is consistent with a radio telemetry study of vizcachas that showed that puma predation on vizcachas is much higher in summer than in winter and concurrent with the period of vociferous territorial displays (L. Branch, pers. obs.). Livestock occurred at a very low frequency in the diet of pumas (5%) during both seasons, perhaps because pumas can access enough large-bodied native prey all year. Also, herders move many goats out of the northern part of our study area in winter during the time that guanaco densities are low [66].

**Fig 2:**
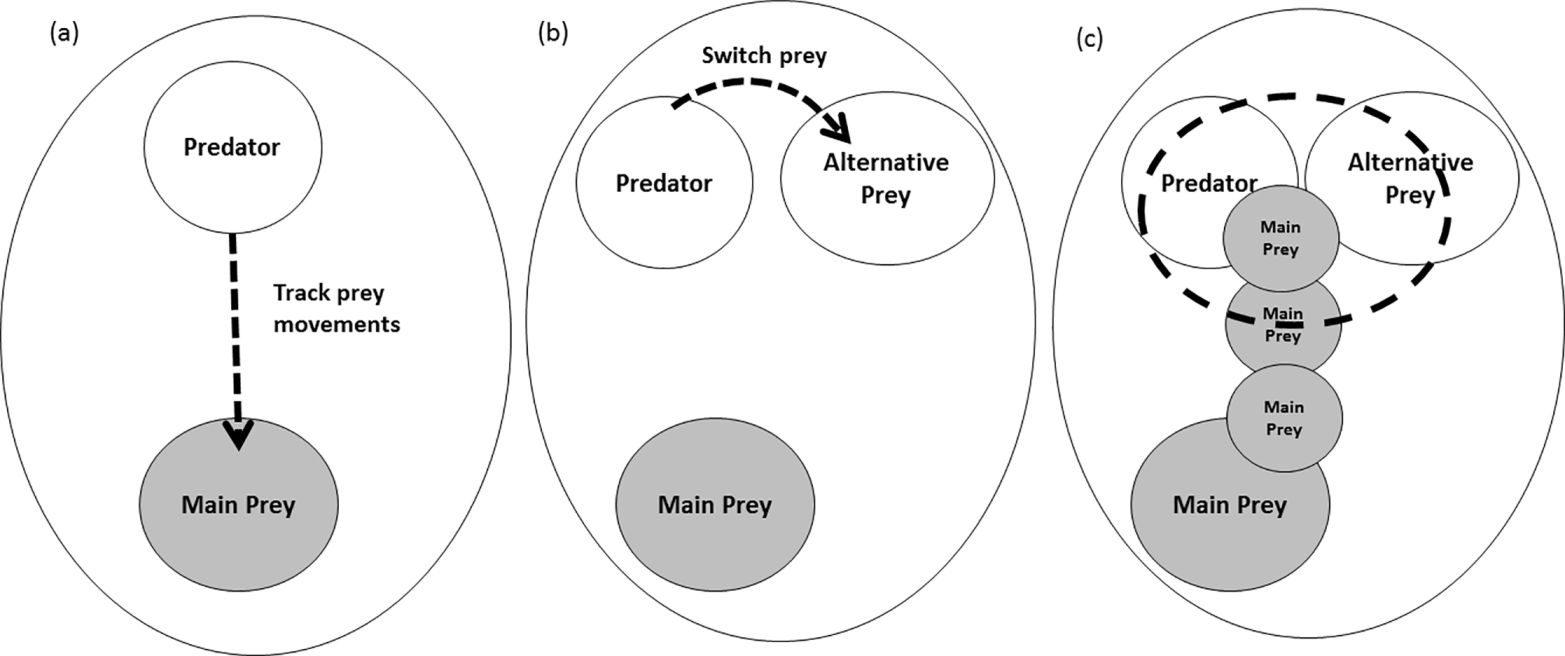
Responses of pumas to seasonal migration of their primary prey. (a) Pumas move with their prey as prey migrate [18], (b) Pumas switch to alternative prey when their primary prey migrate [14], (c) Pumas consume residual non-migratory individuals of their primary prey species and alternative prey when most of their primary prey migrate (this study).

Although prey migration, including partial migration, is widespread across the geographic distribution of pumas [12,18,26,36,67], data on responses of pumas to migratory prey are still scarce. Our data combined with those of other studies indicate that pumas exhibit at least 3 responses when prey migrate. They rely on non-migratory individuals of the primary prey species and alternative prey, as documented in this study, switch to alternative prey, or follow the prey migration (Fig 2). Similar to our study, pumas in the Southern Yellowstone Ecosystem, Wyoming, remain relatively stationary during seasonal migrations of their ungulate prey [14]. However in Yellowstone, entire prey populations migrate, but pumas have the option of feeding on a variety of ungulates and switch prey as prey densities change with migration. In contrast, in the eastern Sierra Nevada, California, pumas move seasonally with their primary prey, mule deer (*Odocoileus hemionus;* [18]). More than 99% of the deer in this area migrate, and the only other large-bodied prey are bighorn sheep (*Ovis canadensis*), which occur in small populations; thus, puma populations appear to be limited by food [68–70]. The breadth of responses of pumas to migratory prey is consistent with flexibility in other aspects of their biology such as diet [28,63] and habitat use [71,72] that likely contribute to the ability of this species to occupy the majority of two continents.

Given that pumas fed primarily on guanacos throughout the year, abundance of resident guanacos could limit puma densities in the north part of the study area during the winter and the south part of the study area during summer. However, other factors such as vulnerability of prey and human-induced mortality of pumas also likely are important. Although puma densities did not change in parallel with guanaco migration, consistent spatial trends occurred in puma density, though confidence intervals were large. Density of pumas was greater in the northern part of our study area compared to the south in both winter and summer. The north has large volcanos, more rocky outcrops, and higher vegetation cover than the open plains of the south [73] and may provide more refuges and cover for stalking prey and, thus, higher habitat quality for pumas. Puma predation on guanacos is higher in areas dominated by rocky escarpments than in open habitat in other parts of La Payunia Reserve [24]. Another potential explanation for spatial differences in puma density in the reserve is that human-induced mortality of pumas may be greater in the south. This area includes large amounts of private land and is poorly protected by park guards compared to the northern part of the reserve.

Density estimates for pumas obtained in this study are similar to estimates from much of western North America (Table 3), even though density estimates in these studies were based on different methods (e.g., capture-recapture and telemetry studies [34,74,75]). Our estimates of puma density in northern La Payunia, the part of the reserve with highest protection, are higher than estimates from further south in Patagonia [60,76], the Mediterranean Andes [77], and sites in northern Argentina with high poaching pressure (Atlantic Forest and Chaco, [39,40]; Table 3), but closer to estimates for sites with low poaching pressure in the Atlantic Forest and semiarid Calden Forest in the province neighboring La Payunia [33,76]. Even though poaching occurs in La Payunia, this threat likely is lower than in most of the Atlantic Forest and Argentine Chaco, which have much larger human populations as well as higher habitat loss and degradation [39, 78,79].

**Table 3:** Puma density estimates from other sites in Central and South America and sites in western North America with shrublands and grasslands as in La Payunia Reserve, Argentina.

Country	Habitat	Adult pumas/ 100 km^2^	Method	Reference
Argentina				
La Pampa	Semiarid Calden forest			
	Ranch with sport hunting	1.4^c^	CT	[41]
	Provincial park	4.9^c^	CT	[41]
Misiones	Subtropical Atlantic forest			
	High poaching pressure	0.3^c^-0.8^a^	CT	[32,39]
	Low poaching pressure	2.2^a^	CT	[54]
Santiago del Estero and Formosa	Semiarid Chaco forest	0.2–1.1^a^, 0.1–1.3^b^	CT	[40]
Bolivia				
Santa Cruz	Dry Chaco forest	6.5^c^- 8.0^a^	CT	[32,37]
Belize	Tropical forest	0.6^c^- 4.9^a^	CT	[32,39]
Brazil				
Tocantins and Pará	Tropical forest	3.4^a^	CT	[49]
Chile				
Aysén (XI Region)	Patagonian steppe and forest	1.3^d^	RT	[60]
Magallanes (XII Region)	Patagonian steppe and grassland	2.5^e^	RT	[76]
O’ Higgins (VI Region)	Mediterranean Andes	0.7^a^	CT	[77]
Mexico				
State of Mexico	Mountain forest and grassland	1.2–6.9^a^	CT	[43]
United States				
Idaho	Ponderosa pine and desert shrublands	1.7–3.5^d^	RT	[74]
New Mexico	San Andres Mountains, Chihuahuan Desert	1.7–3.9^d^	RT	[29]
Utah	Pinyon pine and desert shrublands	0.6–1.4^d^	RT	[80]
Utah	Colorado Plateau and Great Basin Desert	1.1–3.2^d^	RT	[81]
Wyoming	Ponderosa pine and desert grass and shrublands	3.5–4.6^d^	RT	[75]

Studies were conducted using camera trapping (CT) or radio telemetry (RT). Analysis employed

^a^non-spatial capture-recapture models

^b^SECR

^c^SMR

^d^Home range analysis

^e^Minimum density estimates based on known radio-collared individuals.

In addition to increasing understanding of the role of migration in predator-prey dynamics in this system, baseline data on densities of pumas are important for conservation and management. Pumas are heavily controlled outside protected areas in Patagonia because of their impact on livestock, and sport hunting occurs in some regions of Argentina [41,82]. For elusive species such as pumas, obtaining density estimates can be challenging, particularly in non-forested habitats that lack trails and roads to help guide animals between cameras. To date, only a few studies have used spatial mark-resight models to estimate densities for pumas (e.g., [32,41,83]. However, comparisons of approaches for estimating density in tropical and subtropical forests demonstrated that traditional non-spatial approaches likely over estimate densities and that a mark-resight framework increases precision compared to other analyses [32]. Although obtaining sufficiently large samples for robust density estimates still remains a challenge, our study demonstrates that the combination of a camera-trapping design that incorporates landscape features to enhance detectability and spatial-mark resight models is a promising tool to estimate population numbers for such elusive species in non-forested habitats and provides a foundation for exploring ecological processes such as predator responses to prey migration that occur over large landscapes.

## Supporting information

S1 Fig Index of guanaco counts in the north grid (summer range) and south grid (winter range) using one record per day per camera station. Independent records of guanacos were summed for intervals of 14 days and are presented here for intervals when all camera trap stations (n = 69) were active. Camera set up was completed for the south grid during the first two-week interval. We used one record per day per camera station as an index of guanaco abundance rather than actual counts of guanacos because photographs generally did not capture all members of guanaco groups. Also, aggregation of guanacos changes between seasons [26], and this could influence detection rate by cameras. In summer, social groups of guanacos comprise family groups, solitary males, and all male groups spread across the landscape. In winter, most guanacos aggregate in much larger mixed groups, which could bias our winter counts low in both grids. Schroeder et al. [23] also documented guanaco migration in the northern part of La Payunia with ground surveys of guanacos along transects. The eastern part of their study area overlaps with our north grid. Similar to our results for this grid, they recorded low counts of guanacos in winter and high counts in summer.
(PDF)

S2 Fig Plots generated by Spatial Mark-Resight to determine appropriate buffer size. A) Plot of estimated density (y-axis, in individuals per hectare) versus buffer size (x-axis) for the best fitting multi-session SMR model. Note the asymptote in density well before reaching the buffer size of 12,000 m used in our analysis.
B) Encounter rate of pumas as a function of distance from the center of a home range for the best-fitting SMR model. Note that encounter rate is near 0 prior to reaching our buffer size of 12,000 m.
(PDF)

S1 Table Model comparison table of *a priori* spatial mark-resight models used to estimate puma density. See text for explanation of covariates. AICc = Akaike’s Information Criterion adjusted for small sample sizes (AICc); Δ AICc = difference between the model AICc and the lowest AICc; λ0 = encounter rate, σ = spatial scale parameter that relates decline in detection with distance from the animal’s activity center; K = number of parameters in the model.
(PDF)

S1 Appendix Spanish abstract (Resumen en español). (PDF)
