# Supplementary figures and images for: Response of pumas (Puma concolor) to migration of their primary prey in Patagonia
Source: PLoS One. 2017 Dec 6;12(12):e0188877. doi: 10.1371/journal.pone.0188877 (PMC5718558; doi:10.1371/journal.pone.0188877)

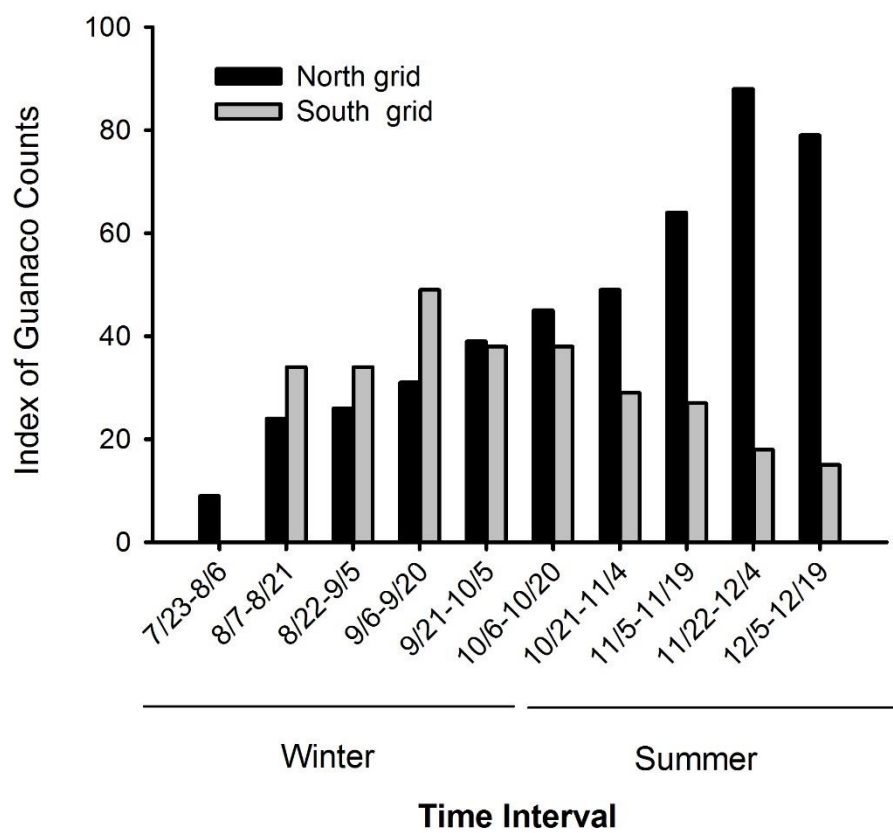

Supplement: S1 Fig — Independent records of guanacos were summed for intervals of 14 days and are presented here for intervals when all camera trap stations (n = 69) were active. Camera set up was completed for the south grid during the first two-week interval. We used one record per day per camera station as an index of guanaco abundance rather than actual counts of guanacos because photographs generally did not capture all members of guanaco groups. Also, aggregation of guanacos changes between seasons [26], and this could influence detection rate by cameras. In summer, social groups of guanacos comprise family groups, solitary males, and all male groups spread across the landscape. In winter, most guanacos aggregate in much larger mixed groups, which could bias our winter counts low in both grids. Schroeder et al. [23] also documented guanaco migration in the northern part of La Payunia with ground surveys of guanacos along transects. The eastern part of their study area overlaps with our north grid. Similar to our results for this grid, they recorded low counts of guanacos in winter and high counts in summer. (PDF) [file pone.0188877.s001.pdf]

A)

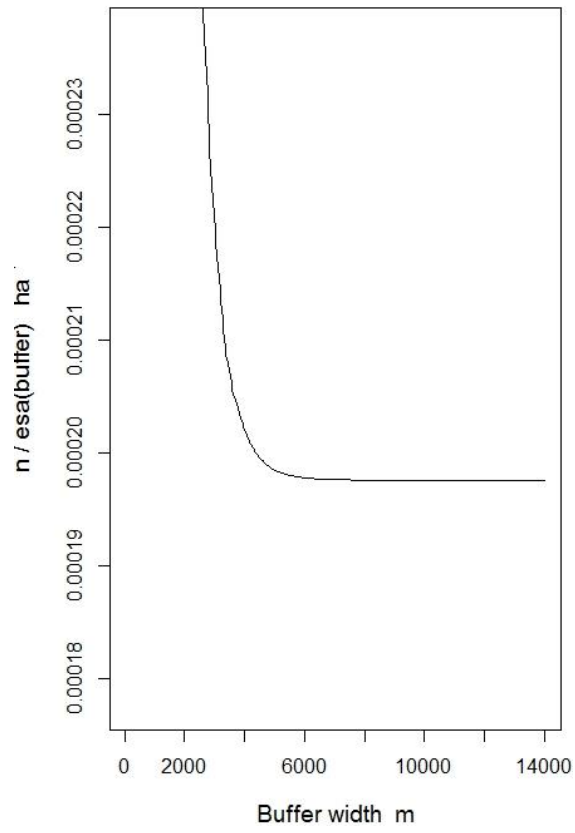

B)

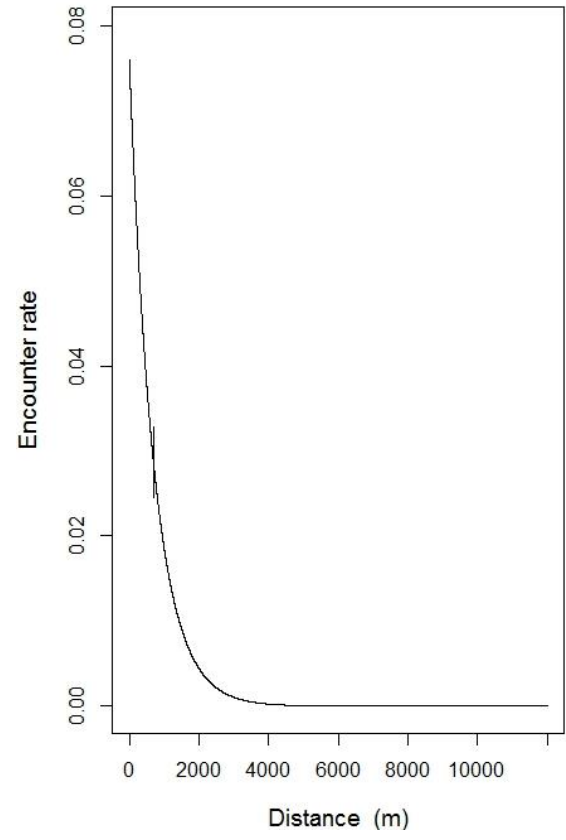

Supplement: S2 Fig — A) Plot of estimated density (y-axis, in individuals per hectare) versus buffer size (x-axis) for the best fitting multi-session SMR model. Note the asymptote in density well before reaching the buffer size of 12,000 m used in our analysis. B) Encounter rate of pumas as a function of distance from the center of a home range for the best-fitting SMR model. Note that encounter rate is near 0 prior to reaching our buffer size of 12,000 m. (PDF) [file pone.0188877.s002.pdf]
