# Supplementary material for: Response of pumas (Puma concolor) to migration of their primary prey in Patagonia
Source: PLoS One. 2017 Dec 6;12(12):e0188877. doi: 10.1371/journal.pone.0188877 (PMC5718558; doi:10.1371/journal.pone.0188877)
Supplement: S1 Table — See text for explanation of covariates. AICc = Akaike’s Information Criterion adjusted for small sample sizes (AICc); Δ AICc = difference between the model AICc and the lowest AICc; λ0 = encounter rate, σ = spatial scale parameter that relates decline in detection with distance from the animal’s activity center; K = number of parameters in the model. (PDF) [file pone.0188877.s003.pdf]

Model	AICc	Δ AICc	λ0	σ	K
D(session) λ0(site), σ(.)	1432	0	0.019	754	7
D(session) λ0(t), σ(.)	1488	56	0.016	805	16
D(session) λ0(.), σ(.)	1512	80	0.027	1273	6
D(session) λ0(k), σ(.)	1647	215	0.000008	1935	7
D(session) λ0(b), σ(.)	1883	451	0.006	773	7
D(.) λ0(site), σ(.)	1532	0	0.017	771	4
D(.) λ0(.), σ(.)	1551	19	0.054	727	3
D(.) λ0(t), σ(.)	1585	53	0.016	838	13
D(.) λ0(b), σ(.)	1934	402	0.007	772	4
D(.) λ0(k), σ(.)	2196	664	0.000007	1713	4
