# Supplementary material for: Response of pumas (Puma concolor) to migration of their primary prey in Patagonia
Source: PLoS One. 2017 Dec 6;12(12):e0188877. doi: 10.1371/journal.pone.0188877 (PMC5718558; doi:10.1371/journal.pone.0188877)
Supplement: S1 Appendix — (PDF) [file pone.0188877.s004.pdf]

Las migraciones de ungulados a gran escala provocan cambios en la disponibilidad de presas para los depredadores tope y, como consecuencia, pueden alterar el comportamiento de éstos depredadores. La migración puede ser de la población completa de las especies presa, pero a menudo las poblaciones de presas muestran una migración parcial con algunos individuos que permanecen residentes y otros que migran. En América del Norte y otras partes del mundo, las interacciones entre presas migratorias y depredadores han sido bien documentadas, en cambio, en América del Sur, éstas han sido escasamente estudiadas. Examinamos la respuesta del puma *(Puma concolor)* a la migración estacional del guanaco *(Lama guanicoe)* en la Reserva Provincial La Payunia al norte de la Patagonia argentina, donde ocurre la migración de ungulados de mayor escala en América del Sur. Mas de 15.000 guanacos migran estacionalmente en la reserva, y algunos son residentes a lo largo del año. Hipotetizamos que los pumas responden a la migración de los guanacos consumiendo más presas alternativas en lugar de migrar con los guanacos, debido a la territorialidad de los pumas y a la disponibilidad de presas alternativas en el sitio a lo largo del año. Para determinar si los pumas se movieron estacionalmente con los guanacos, colocamos cámaras trampa en los sitios de distribución de verano e invierno de los guanacos en ambas estaciones y estimamos la densidad de pumas mediante modelos espaciales de marcado- revisualización espacialmente explícitos (SMR, siglas en inglés). Además, analizamos heces de puma para evaluar cambios en el consumo de presas en respuesta a la migración de los guanacos. Las estimaciones de densidad de pumas no cambiaron significativamente en los sitios de distribución de verano e invierno de los guanacos, cuando éstos migraron hacia y desde éstas áreas, indicando que los pumas no siguen la migración de los guanacos. Además, los pumas no consumieron más presas alternativas y/o ganado cuando la disponibilidad de guanacos disminuyó, sino que incorporaron principalmente guanacos y unas

pocas presas alternativas en su dieta durante ambas estaciones. Esta respuesta difiere a la de otros sitios al oeste de los Estados Unidos, donde las poblaciones completas de presas migran y los pumas siguen a sus presas o modifican su dieta por presas más abundantes cuando su presa principal migró.
